# Retraction Note: *The HOTAIRM1/miR-107/TDG* axis regulates papillary thyroid cancer cell proliferation and invasion

**DOI:** 10.1038/s41419-024-06556-2

**Published:** 2024-02-21

**Authors:** Dan Li, Li Chai, Xiaqing Yu, Yingchun Song, Xuchao Zhu, Suyun Fan, Wen Jiang, Tingting Qiao, Junyu Tong, Simin Liu, Lihong Fan, Zhongwei Lv

**Affiliations:** 1 Department of Nuclear Medicine, Shanghai Tenth People’s Hospital, School of Medicine, Tongji University, 200072 Shanghai, China; 2 Department of Respiratory Medicine, Shanghai Tenth People’s Hospital, School of Medicine, Tongji University, 200072 Shanghai, China

Retraction to: *Cell Death and Disease* 10.1038/s41419-020-2416-1, published online 08 April 2020

The Editors-in-Chief have retracted this article. After publication, concerns were raised regarding the data presented in the figures, specifically:

- Fig. 3a and 6f top right images appear to overlap.
- In Fig. 3c, two images appear highly similar to Fig. 3a in [1], and one image appears highly similar to Fig. 4a in [2].
- Fig. 7f TPC-1 TDG lane 1 appears to stand out from the image background.
- Fig. 7j TPC-1 GAPDH lanes appear to be misaligned vertically.

In addition, the Ethics approval numbers are not provided in the text.

The Editors-in-Chief therefore no longer have confidence in the presented data.

The authors have not responded to any correspondence from the editor or publisher about this retraction.
